# Ran GTPase promotes cancer progression via Met receptor-mediated downstream signaling

**DOI:** 10.18632/oncotarget.12420

**Published:** 2016-10-03

**Authors:** Hiu-Fung Yuen, Ka-Kui Chan, Angela Platt-Higgins, El-Habib Dakir, Kyle B. Matchett, Yusuf Ahmed Haggag, Puthen V. Jithesh, Tanwir Habib, Ahmed Faheem, Fennell A. Dean, Richard Morgan, Philip S. Rudland, Mohamed El-Tanani

**Affiliations:** ^1^ Center for Cancer Research and Cell Biology, Queen's University Belfast, Belfast, UK; ^2^ Cancer and Polio Research Fund Laboratories, School of Biological Sciences, University of Liverpool, Liverpool, UK; ^3^ Translational Clinical Research, University of Leicester, Leicester, UK; ^4^ Institute of Cancer Therapeutics, University of Bradford, Bradford, West Yorkshire, UK; ^5^ University of Sunderland, Department of Pharmacy, Health and Well-Being, Sunderland Pharmacy School, Sunderland, UK; ^6^ Biomedical Informatics Research, Sidra Medical and Research Center, Doha, Qatar; ^7^ Department of Pharmaceutical Technology, Faculty of Pharmacy, University of Tanta, Tanta, Egypt

**Keywords:** Ran GTP, C-Met, breast cancer, lung cancer, gefitinib

## Abstract

It has been shown previously that cancer cells with an activated oncogenic pathway, including Met activation, require Ran for growth and survival.

Here, we show that knockdown of Ran leads to a reduction of Met receptor expression in several breast and lung cancer cell lines. This, in turn suppressed HGF expression and the Met-mediated activation of the Akt pathway, as well as cell adhesion, migration, and invasion. In a cell line model where Met amplification has previously been shown to contribute to gefitinib resistance, Ran knockdown sensitized cells to gefitinib-mediated inhibition of Akt and ERK1/2 phosphorylation and consequently reduced cell proliferation. We further demonstrate that Met reduction-mediated by knockdown of Ran, occurs at the post-transcriptional level, probably via a matrix metalloproteinase. Moreover, the level of immunoreactive Ran and Met are positively associated in human breast cancer specimens, suggesting that a high level of Ran may be a pre-requisite for Met overexpression. Interestingly, a high level of immunoreactive Ran dictates the prognostic significance of Met, indicating that the co-overexpression of Met and Ran may be associated with cancer progression and could be used in combination as a prognostic indicator.

## INTRODUCTION

Ran GTPase (Ran) is a Ras-related protein that is involved in cell cycle regulation, nuclear-cytoplasmic transportation, and cell transformation [1, 2]. Recently, we and others have shown that Ran plays an important role in cancer cell survival and cancer progression [3–5]. Upstream factors that drive Ran expression and activity, and hence cause enhanced cell proliferation, cell survival, and cancer progression, have recently been identified and studied. These include the Ras and PI3K pathways [6], oesteopontin [7], RASSF1A [8], Sgk1 [9, 10] and Myc [11]. However, less is known of the downstream effectors of Ran.

The Met receptor has been shown to play an important role in both breast [12] and lung [13, 14] cancer. Cancer cells with Met amplification are addicted to Met for growth and survival [15]. Met overexpression is associated with resistance to trastuzumab and gefitinib in breast [16, 17] and lung [18, 19] cancer cells, respectively. Recently, Met overexpression has been shown to be associated with basal breast cancer [17, 20, 21], the most aggressive subtype of breast cancer, while chemical or biological inhibition of Met has been shown to reduce breast cancer-derived bone metastasis [20]. In lung cancer, Met is a potential therapeutic target, and inhibitors of its tyrosine kinase activity together with anti-Met antibodies are being investigating in clinical trials [22, 23]. Indeed, Met expression has been shown to play an important role in other cancer type such as hepatocellular carcinoma [24].

Previously, a Ran binding protein, RanBPM, was shown to promote HGF-Met signaling [25], and we have shown that, in a rat cell line, Ran activates Met to promote cancer progression [7]. Nevertheless, to the best of our knowledge, there is no report describing the role of Ran in HGF-Met-mediated signaling in the progression of human cancer. Here, we show that Ran knockdown results in the reduction of Met, via post-transcriptional regulation, resulting in reduced responsiveness towards HGF-stimulated biological properties associated with cancer progression, and sensitization of cells to gefitinib treatment.

## RESULTS

### Knockdown of Ran results in the down-regulation of Met in multiple cancer cell lines

**Figure 1:**
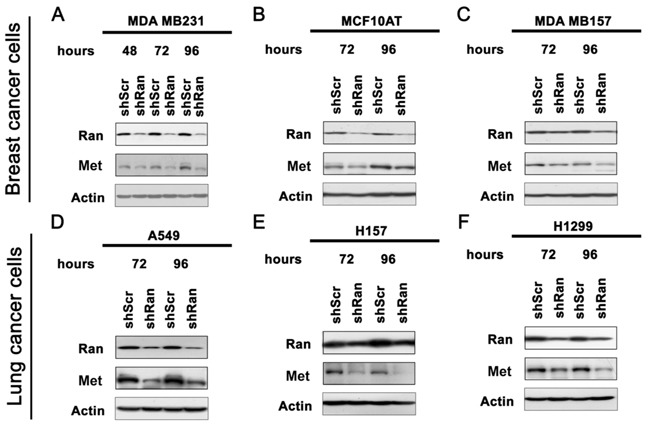
Knockdown of Ran downregulates Met in multiple cell lines Western blot for Ran, Met and Actin in breast cancer cell lines **A.** MDA MB231, **B.** MCF10AT and **C.** MDA MB157, and lung cancer cell lines **D.** A549, **E.** H157 and **F.** H1299.

Previously, we have shown that knockdown of Ran using potent shRNA results in apoptosis [5]. To study the effect of Ran knockdown on cell properties without interference by apoptosis, a less potent shRNA was used, which was previously shown not to induce appreciable apoptosis even at 96 hours post-transfection, despite a significant reduction (~60%) in Ran expression [5]. Using this shRNA, we now find that knockdown of Ran in breast cancer cell lines MDA MB231 (Figure 1A), MCF10AT (Figure 1B) and MDA MB157 (Figure 1C) and lung cancer cell lines (A549 (Figure 1D), H157 (Figure 1E) and H1299 (Figure 1F)) resulted in down-regulation of Met protein.

### Knockdown of Ran reduces the responsiveness of cancer cells to HGF-induced phosphorylation of Met and Akt, but not of ERK1/2

**Figure 2:**
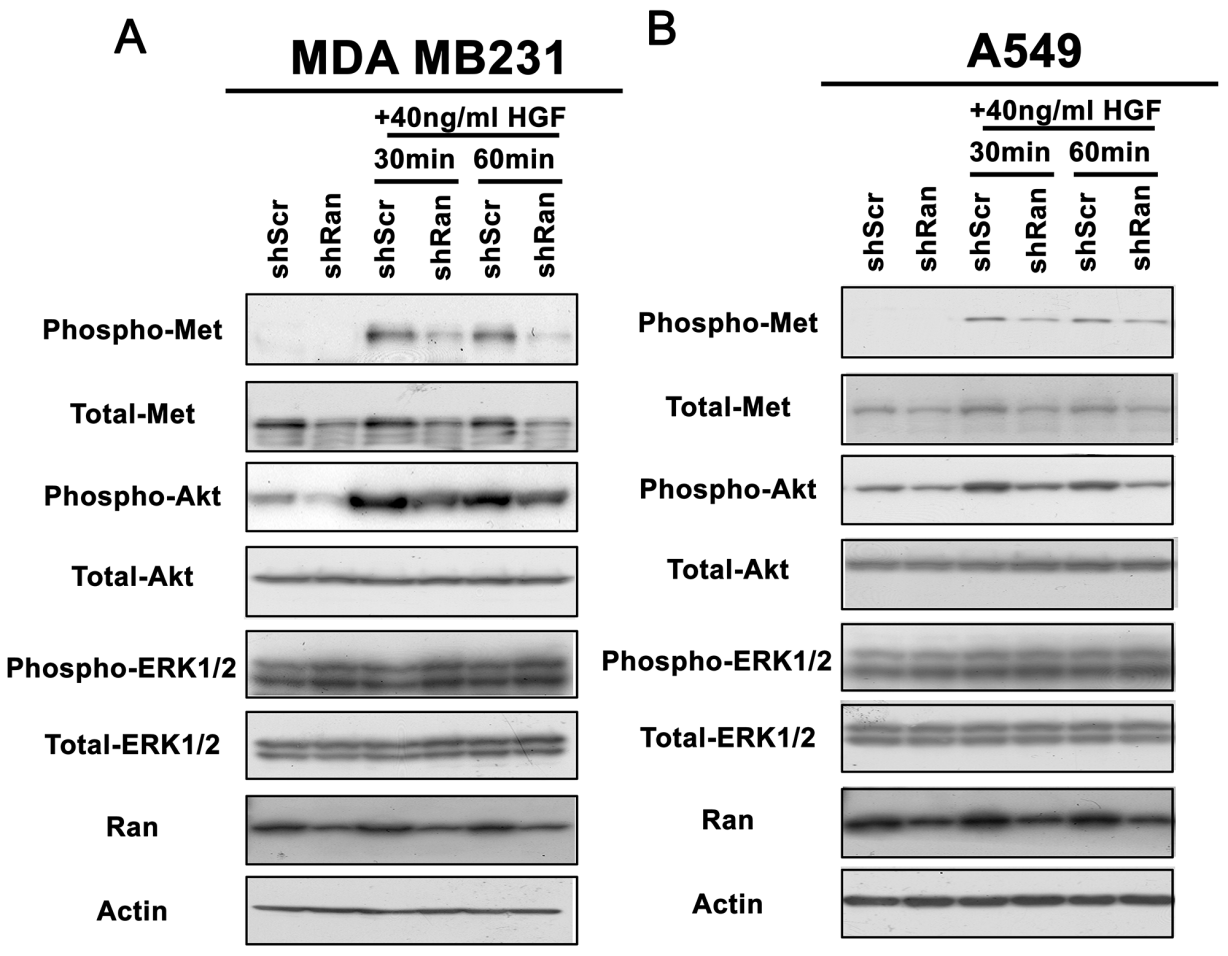
Knockdown of Ran reduces responsiveness of Met-Akt signalling to stimulation by HGF Western blot for phospho-Met, total-Met, phospho-Akt, total-Akt, phospho-ERK1/2, total-ERK1/2, Ran, and Actin for **A.** MDA MB231 and **B.** A549 cells cultured in serum-free conditions in the presence or absence of HGF for 30 or 60 mins, 48 hour post-infection with shScr or shRan. Note that both Met and Akt phosphorylation were more prominently reduced by Ran knockdown in the presence of 10 ng/ml HGF in both cell lines.

As Met expression was reduced upon Ran knockdown, we next investigated whether this reduced Met signaling in both breast and lung cancer cell lines. Knockdown of Ran in both MDA MB231 breast and A549 lung cancer cells resulted in a reduction of the Met receptor in serum-free conditions (Figure 2). Treatment of cells with HGF for 30 – 60 minutes resulted in a dramatic induction of phosphorylation of Met and Akt but not of ERK1/2 (Figure 2). In contrast, Ran knockdown reduced the HGF-mediated phosphorylation of Met and Akt in both cell lines (Figure 2). Again, phosphorylation of ERK1/2, which was not affected by HGF treatment, was not altered by Ran knockdown in the presence or absence of HGF in both cell lines (Figure 2). This result suggests that Ran knockdown specifically reduced the responsiveness towards HGF in terms of Met signaling in both breast (Figure 2A) and lung (Figure 2B) cancer cell lines.

### Knockdown of Ran reduces the responsiveness of cancer cells to HGF-induced cell adhesion, migration, and invasion

**Figure 3:**
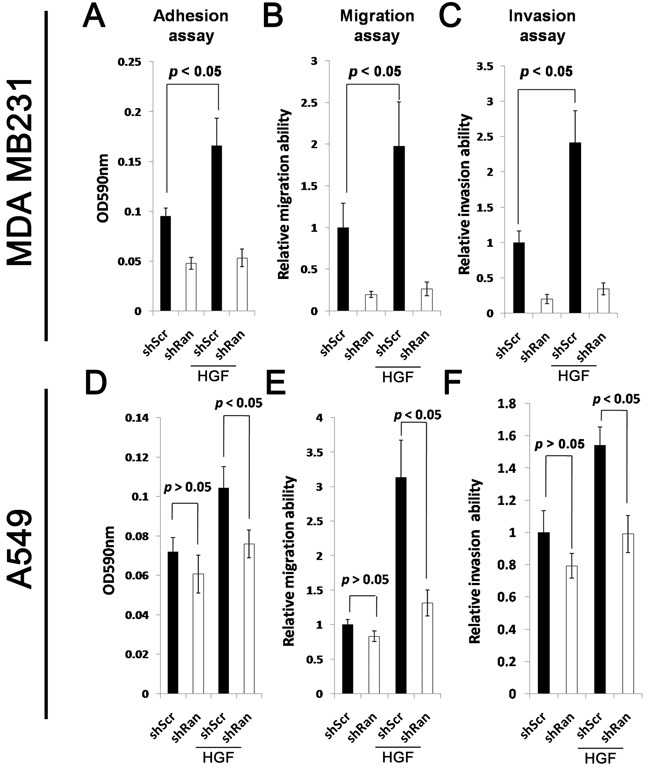
Knockdown of Ran reduces responsiveness of HGF-stimulated cell adhesion, migration, and invasion **A-C.** MDA MB231 breast cancer cells transfected with either shScr or shRan were suspended in serum-free conditions in the presence or absence of 10 ng/ml HGF for the (A) adhesion, (B) migration, and (C) invasion assays. **D-F.** A549 lung cancer cells infected with either shScr or shRan were suspended in serum-free conditions in the presence or absence of HGF for the (A) adhesion, (B) migration, and (C) invasion assay.

Activation of Met signaling promotes invasive cancer growth [26, 27]. We therefore evaluated whether reduced Met signaling upon Ran knockdown causes a reduction in the aggressiveness of the cancer cells. HGF treatment of MDA-MB-231-shScr control cells resulted in a significant increase in cell adhesion (Figure 3A; Student's t test at *p* < 0.05), migration (Figure 3B; *p* < 0.05), and invasion (Figure 3C; *p* < 0.05). In contrast, treatment of Ran knockdown MDA MB231-shRan cells with HGF did not significantly alter cell adhesion (Figure 3A); Student's t test p < 0.05, migration (Figure 3B), or invasion (Figure 3C). Similarly, HGF treatment of lung cancer derived A549-shScr cells, but not A549-shRan cells, resulted in a significant increase in cell adhesion (Figure 3D; *p* < 0.05), migration (Figure 3E; *p* < 0.05), and invasion (Figure 3F; *p* < 0.05). Interestingly, Ran knockdown did not alter the cellular properties of A549 cells in the absence of HGF, but did so in the presence of HGF (p<0.05) (Figure 3D–3F), suggesting that knockdown of Ran led to reduced cell adhesion, migration, and invasion only when Met signaling was activated. Collectively, our results suggest that Ran knockdown reduces the Met signaling-induced invasive properties of cancer cells *in vitro*.

### Knockdown of Ran diminishes Met overexpression-mediated gefitinib resistance

**Figure 4:**
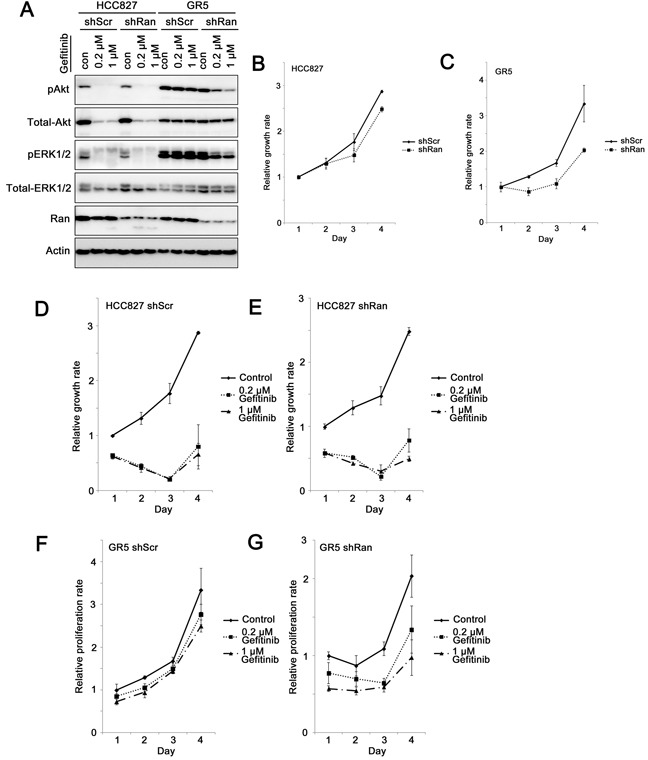
Knockdown of Ran sensitizes Met-overexpressing gefitinib-resistant lung cancer cells to gefitinib **A.** Western blot for phospho-Akt, total-Akt, phospho-ERK1/2, total-ERK1/2, Ran, and Actin for HCC827 parental and GR5 cells in the presence or absence of 0.2 μM or 1 μM gefitinib. **B-G.** MTT growth assay for (B) HCC827 parental and (C) HCC827 GR5 cells infected with either shScr or shRan, (D) HCC827-shScr, (E) HCC827-shRan, (F) HCC827 GR5-shScr, and (G) HCC827 GR5-shRan cells with or without treatment with 0.2 μM or 1 μM gefitinib.

Met overexpression in HCC827 GR5 lung cancer cells renders them insensitive to gefitinib-mediated inhibition of the PI3K/Akt and MEK/ERK pathways and gefitinib-induced apoptosis compared to HCC827 parental cells [18]. Here, we investigated whether Ran knockdown sensitizes Met-overexpressing GR5 cells to gefitinib treatment. Gefitinib treatment of both HCC827-shScr and HCC827-shRan inhibited Akt and ERK1/2 phosphorylation (Figure 4A). In contrast in GR5-shScr cells, phosphorylation of Akt and ERK1/2 was not significantly altered in the presence of up to 1 μM gefitinib (Figure 4A). However when Ran was knocked down in GR5-shRan cells, reduction in phosphorylation of both pAkt and pERK1/2 was observed in the presence of geftinib (Figure 4A). These results suggest that Ran knockdown sensitizes GR5 cells to gefitnib-mediated inhibition of both the PI3K/Akt and MEK/ERK pathways.

Similar to our previous study [5], when mediated by this less potent shRNA, knockdown of Ran did not alter the growth rate rate of HCC827 parental cells (shScr vs. shRan, Games-Howell post-hoc test, *p* = 0.158; Figure 4B), but reduced the growth rate of HCC827 GR5 cells (shScr vs. shRan, Games-Howell post-hoc test, *p* = 0.044; Figure 4C). More importantly, the difference in the growth rate between the parental and GR5 cells and between shScr vs shRan was statistically significantly (*p* = 0.01), indicating that cancer cells with Met overexpression are more sensitive to Ran down-regulation.

HCC827 lung cancer cells were highly sensitive to gefitinib treatment, as both shScr (control vs. gefitinib, Games-Howell post-hoc test, *p* = 0.002 and *p* = 0.001, for 0.2 μM gefitinib and 1 μM gefitinib, respectively; Figure 4D) and shRan transfected (control vs. gefitinib, Games-Howell post-hoc test, *p* = 0.002 and *p* = 0.001, for 0.2 μM gefitinib and 1 μM gefitinib, respectively; Figure 4E) HCC827 cells showed a significant reduction in growth upon gefitinib treatment. In contrast, HCC827 GR5 was resistant to gefitnib treatment, with no significant inhibition of growth following exposure of GR5-shScr cells to 1 μM gefitinib (control vs. gefitinib, Games-Howell post-hoc test, *p* = 0.461 and *p* = 0.227, for 0.2 μM gefitinib and 1 μM gefitinib, respectively; Figure 4F). In contrast, knockdown of Ran in HCC827-GR5 cells resulted in their sensitization to gefitinib. Treatment of GR5-shRan cells with gefitinib resulted in a significant reduction in growth (control vs. gefitinib, Games-Howell post-hoc test, *p* = 0.167 and *p* = 0.008, for 0.2 μM gefitinib and 1 μM gefitinib, respectively; Figure 4G). The interaction among cell lines (HCC827 parental vs. GR5), shRNAs (shScr vs. shRan) and treatment (control vs. 1μM gefitinib) was statistically significant (*p* = 0.048; Figure 4D-G), suggesting that Ran knockdown increases the sensitivity of HCC827-GR5 cells, but not HCC827 parental cells to gefitinib treatment.

### Reduction of Met expression by Ran knockdown involves a post-transcriptional step

**Figure 5:**
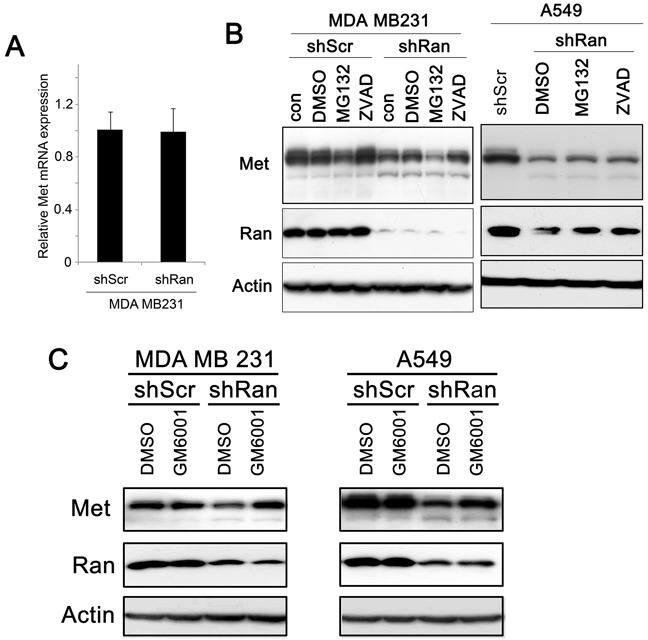
Ran knockdown-mediated Met downregulation occurs post-transcriptionally **A.** Real-time PCR of Met in MDA MB231-shScr and MDA MB231-shRan cells. **B.** Western blot for Met, Ran, and Actin in MDA MB231 breast (Left) in A549 lung (Right) cancer cells transfected with either shScr or shRan with or without treatment with DMSO as control vehicle, MG132 (Proteasome inhibitor) 10 μM and Caspase Inhibitor Z-VAD-FMK (20μM). **C.** Western blot for Met, Ran, and Actin in MDA MB231 breast (Left) and in A549 lung (Right) cancer cells infected with either shScr or shRan or GM6001 (a metalloprotease inhibitor).

To investigate how Ran knockdown contributes to a reduction in Met expression, we first investigated whether it occurs at a transcriptional level. Using real-time PCR, we found that levels of Met mRNA were not significantly different between MDA MB231-shScr and MDA MB231-shRan cells (Figure 5A). In other systems Met expression was previously shown to be regulated at the post-transcriptional level by either caspases [28, 29], via proteasome degradation [30, 31], or by metalloproteinase digestion [32, 33]. In the present study, we found the reduction in Met expression by Ran knockdown was not diminished by ZVAD and MG132 treatment in both breast (MDA MB231; Figure 5B, left panel) and lung (A549; Figure 5B, right panel) cancer cell lines, indicating that caspase and proteasome degradation were not involved in the reduction in levels of Met by Ran In contrast, we found that treatment with GM6001, a metalloproteinase inhibitor, counteracted the downregulation of Met levels upon Ran knockdown in both MDA MB231 (Figure 5C, left panel) and A549 (Figure 5C, right panel) cancer cells. Collectively, our results suggest that the Ran knockdown-mediated reduction in Met expression occurs at the post-transcriptional level, and probably involves a metalloproteinase.

### Relationship between Ran and Met expression in human breast cancer specimens

**Figure 6:**
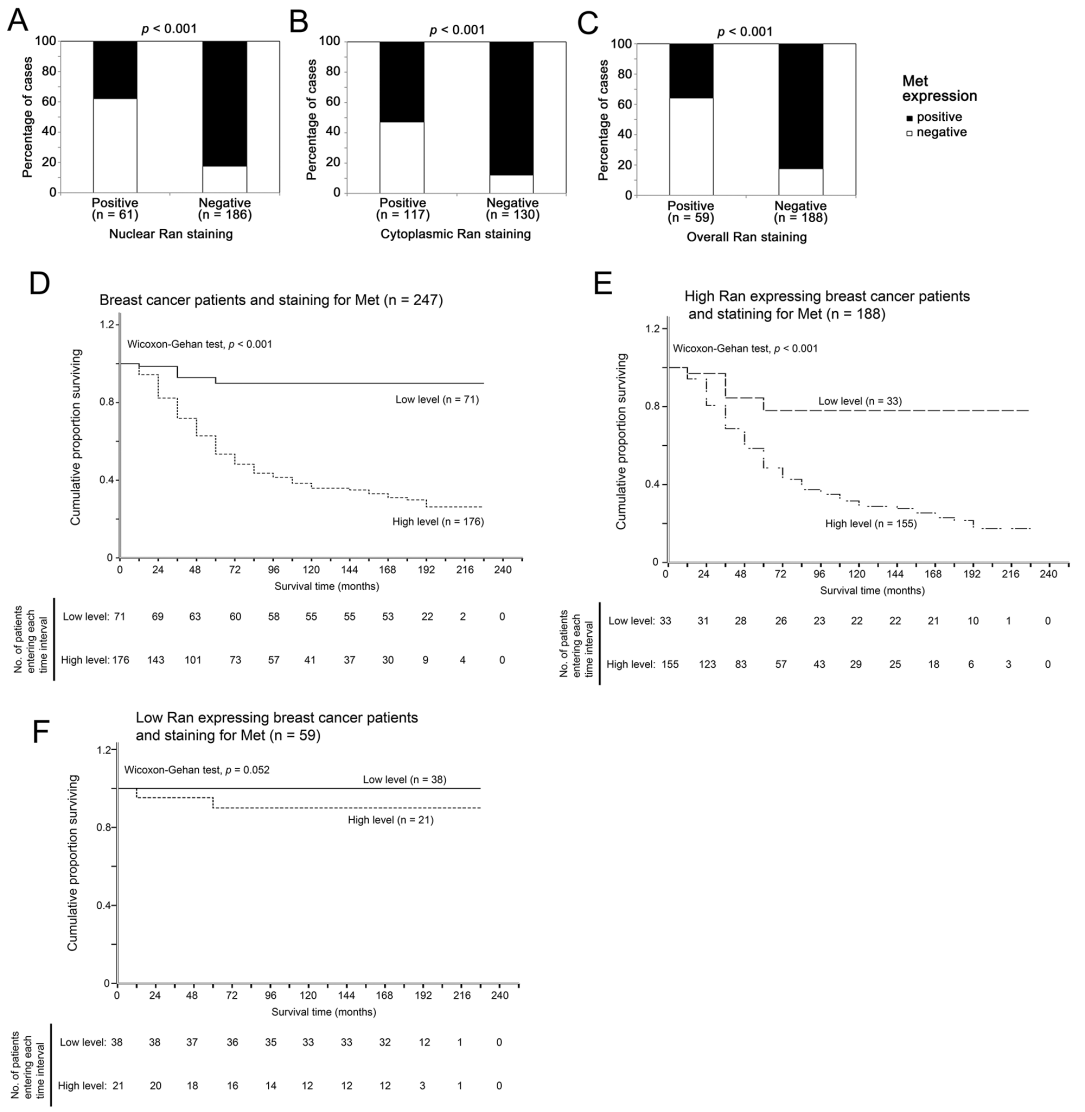
Relationship between tumor staining for Ran and Met tumor survival in breast cancer patients **A-C.** The association between immunohistochemical staining for Ran and Met in human breast cancer specimens. (A) A high level of staining for nuclear Ran in tumors was significantly associated with a high level of Met staining (Fisher's Exact test, *p* < 0.001). (B) A high level of cytoplasmic Ran staining was significantly associated with a high level of staining for Met (Fisher's Exact test, *p* < 0.001). (C) A high level of staining for Ran was significantly associated with a high level of staining for Met when analyzing all specimens (Fisher's Exact test, *p* < 0.001). **D-F.** Relationship between tumor staining for Met and survival time in human breast cancer specimens. (D) Kaplan-Meier plot and wilcoxon-Gehan statistics showed that positive staining for Met expression was significantly associated with a shorter survival time in the breast cancer patient cohort (χ2 = 47.964, 1 df, ρ<0.001). (E) Kaplan-Meier plot and wilcoxon statistics showed that a high level of staining for Met was significantly associated with a shorter survival time in breast cancer patients with positive staining of Ran in the primary tumors (χ2 = 17.307, 1 df, ρ<0.001). (F) Kaplan-Meier plot and Wilcoxon statistics showed staining for Met was not significantly associated with survival time in patients with low Ran expression in the primary tumors (χ2 = 3.774, 1 df, ρ=0.052).

Amongst 247 human breast cancer specimens, 71 contained less than 1% of cancer cells that immunohistochemically stained positive for Met, and 176 specimens contained more than 1% of cancer cells that stained positive for Met. Only 23 out of 61 (37%) nuclear Ran negative specimens stained positive for Met, while significantly more nuclear Ran positive specimens (82%, 153 out of 186) stained positive for Met (Fisher's Exact test, *p* < 0.001; Figure 6A). Similarly, 62 out of 117 (53%) cytoplasmic Ran-negative specimens stained positive for Met, while significantly more cytoplasmic Ran-positive specimens (88%, 114 out of 130) stained positive for Met (Fisher's exact test, *p* < 0.001; Figure 6B). Similar results were obtained when analyzing the association between overall nuclear and cytoplasmic Ran staining and Met staining in these human breast cancer specimens (*p* < 0.001; Figure 6C). Together, our results suggest that Ran expression and Met expression were positively associated in human breast cancer specimens, in agreement with the *in vitro* data presented above.

### Met expression is only associated with shorter survival time in those breast cancer patients with Ran-positive tumors

In the whole breast cancer patient cohort, high immunohistochemical staining for Met was significantly associated with shorter patient survival times (Wilcoxon-Gehan test, *p* < 0.001; Figure 6D). However, when tumors were stratified according to immunohistochemical detection of Ran expression, this association was only significant in patients with a high level of immunoreactive Ran expression (Wilcoxon-Gehan test, *p* < 0.001; Figure 6E), and not in patients with low Ran expression (Wilcoxon-Gehan test, *p* = 0.052; Figure 6F).

## DISCUSSION

In the present study, we have shown that Ran knockdown reduces Met protein levels, probably via metalloproteinase. The reduction in Met results in a reduced responsiveness of cancer cells to HGF, including PI3K/Akt activation downstream of Met and cellular properties associated with metastatic potential that are enhanced by HGF stimulation. Met reduction by knockdown of Ran in Met-overexpressing, gefitinib-resistant lung cancer cells sensitizes the cells to gefitinib. Moreover, an association between immunohistochemical staining for Ran and Met was also observed in human breast cancer specimens, and an association between staining for Met and patient survival was only found in patients with tumors showing high levels of immunoreactive Ran. Our findings show for the first time a signalling link between Ran and Met, and the potential role of Ran as a therapeutic target for cancers addicted to Met signaling, including Met resistant cancers.

Previously, we and others have shown that k-Ras mutant [5, 34], PTEN-deleted [5], Met [5], and Myc [11] overexpressing cancer cells are more sensitive to Ran knockdown compared to their wild-type counterparts, suggesting that Ran may be a potential therapeutic target for cancers with these oncogenic mutations. Met receptor is a promising therapeutic target in multiple cancer types [35], especially non-small cell lung cancer, in which Met overexpression has recently been shown to contribute to EGFR TKI resistance, including resistance to gefitinib [18] and erlotinib [36]. To counter this resistance, several agents have been investigated in clinical trials [22] including tivantinib [37], a tyrosine kinase inhibitor, and MetMab [38], a monovalent antibody targeting the Met receptor, both of which are highly anticipated drugs that are currently being tested in clinical trials in lung cancer. In the present study, we have shown that Ran knockdown results in the down-regulation of Met and reduced Met-induced metastatic potential and gefitinib resistance, identifying a novel and additional role for Ran knockdown as a potential therapeutic approach for cancers addicted to Met signaling, including those cancers with acquired resistance to EGFR TKIs.

Although we have shown that Ran knockdown-mediated reduction in Met is a general phenomenon occurring in all 7 of the cell lines tested, including 3 breast and 4 lung cancer cell lines, and that there is an association between Ran and Met expression, it remains unclear how Ran knockdown reduces the level of Met receptor in human breast cancer. A transcriptional process may be involved since Ran regulates transportation of transcription factors into the nucleus [39], and reduced import of a transcriptional activator of Met expression could be a possible explanation for the reduction in Met after Ran knockdown. However, in this study we found that the level of mRNA expression of Met was not different after Ran knockdown, suggesting that the reduction in Met occurs post-transcriptionally. Previous findings in other systems suggested that caspases [28, 29], the proteasome [30, 31], or metalloproteinase [32, 33] may mediate the interaction between Ran and Met. Our results support the last possibility, since inhibition of metalloproteinase by GM6001 restored the level of Met in Ran knock-down MDA MB231 and A549 cells. However, the exact metalloproteinase(s) involved have yet to be identified. Since metalloproteinases can be regulated via enzyme activation and inhibition, complex formation, and compartmentalization [40], it is quite possible that the intermediate metalloproteinases that act(s) between Ran and Met may not be regulated transcriptionally, and the identification of this metalloprotease is important to further our understanding of the interaction between Ran and Met and to provide a further therapeutic target for Ran-related neoplastic disease.

## MATERIALS AND METHODS

### Cell lines, plasmids, transfection, and viral infection

The MDA MB231 and MDA MB157 breast cancer cell lines, the A549 lung cancer cell line (ATCC), and the viral packaging 293T cell line were maintained in DMEM supplemented with 10% (v/v) fetal bovine serum and antibiotics. H157, H1299, HCC827 parental, and the Met overexpressing HCC827-GR5 lung cancer cell lines (ATCC). HCC827-GR5 was gift from Prof Pasi Janneand developed to be resistant to Gefitinib by amplification of Met gene were maintained in RPMI supplemented with 10% (v/v) fetal bovine serum and antibiotics. The Ras-transformed MCF10A derivative MCF10AT cell line (from Karmanos Cancer Centre, Detroit, MI) was maintained in DMEM/F-12 containing 5% horse serum, 10 μg/ml insulin, 20 ng/ml EGF, 100 ng/ml (v/v) choleratoxin, and 0.5 μg/ml hydrocortisone.

Transfection was performed using GeneJuice® (Promega, Southampton, UK) according to the manufacturer's instructions. pLKO.1-shScr and pLKO.1-shRan5 (CCGGCAGTTCAAACTTGTATTGGTTCTCGAGAACCAATACAAGTTTGAACTGTTTTTTG; Sigma-Aldrich, Dorset, UK) were used for to knockdown Ran by Lentiviral infection, as previously described [5].

### Real-time polymerase chain reaction

RNA was extracted using Trizol (Invitrogen, Paisley, UK) and reverse transcription was performed using SuperScript^TM^ III first strand synthesis system (Invitrogen) according to the manufacturer's instructions. Real-time PCR was performed according to the manufacturer's instructions (Applied Biosystem, Foester City, CA) using a Taqman® assay for Met (Hs01565581_m1, Applied Biosystem).

### Western blot analysis

Western blotting was performed as previously described [5]. Briefly, cells were lysed in RIPA buffer containing protease inhibitors, and equal amounts of proteins were loaded onto a SDS-PAGE gel and transferred onto a nitrocellulose membrane (Millipore, Billerica, MA). The proteins of interest were then detected using specific primary antibodies against phospho-Met (Tyr1359), total-Met, phospho-Akt (Tyr 473), total-Akt, phospho-ERK, total-ERK (Cell Signaling, Danvers, MA), and Ran (Millipore) were used at 1:1000 dilution, while anti-Actin (Sigma) was used at 1:10000 dilution.

### MTT growth assay

Cells were seeded at a density of 3000 cells/well in a 96-well plate in triplicate. Cells were allowed to grow for 24 hours and drugs were then applied to the cells. MTT uptake by the cells was measured at 24, 48, 72 and 96 hours post-treatment.

### Boyden chamber migration and invasion assays

Migration and invasion assays were performed as previously described [41]. Briefly, 5000 and 50000 cells in serum-free conditions were seeded into the upper chamber (Millipore) on top of the membrane with or without Matrigel coating, respectively, for migration and invasion assays. The cells were allowed to migrate or invade towards the bottom layer with 10 ng/ml HGF as a chemoattractant for 24 hours. Cells in the bottom of the membrane were fixed and stained with crystal violet.

### Cell adhesion assay

For the cell the adhesion assay, 40000 cells/well in suspension in serum-free conditions with or without 10 ng/ml HGF were seeded in a 96-well plate coated with fibronectin and allowed to settle for 30 min. Suspended cells were removed by washing 4 times with PBS. Adhered cells were fixed and stained with crystal violet. The excess dye was washed out and the retained dye was extracted. The absorbance at 595nm was measured in a microplate reader.

### Patients and breast cancer specimens

Patients and specimens were described previously [5].

### Immunohistochemical staining and evaluation

Immunohistochemical staining and evaluation of the staining was performed as previously described [5].

### Statistical analysis

Statistical analysis was performed using SPSS 19.0 software. Differences in expression levels between groups/samples were analyzed by Fisher's Exact test. Survival analysis was performed using Kaplan-Meier plots and differences were tested using Wilcoxon-Gehan statistics. A p-value of <0.05 was considered significant in all statistical analyses.
